# M. Laennec on Angina Pectoris

**Published:** 1828-04-01

**Authors:** 


### XVII.

*On Nervous Affections of the Heart and Vessels.* By the late M. Laennec.

[Forbes' new Translation.]

As it is our intention shortly to dedicate an article to organic diseases of the heart, and as that article must necessarily be a very extensive one, we take this opportunity of touching on what the illustrious pathologist above-mentioned has denominated "Nervous Affections" of the Central Organs of the Circulation and its great Outlets. M. Laennec justly observes, that the study of pathological anatomy has not been unattended with the disadvantage of blinding a considerable proportion of students and practitioners to every thing but organic lesions—to all affections of the nerves—to all changes in the fluids. "Nevertheless, (says he,) we are bound to admit, that every disease in which we can discover no constant lesion of the solids, nor evident alteration in the fluids, must consist in some disorder of the nervous influence." Of this class are several cardiac and arterial affections, which we are now to notice.

### I. Neuralgia of the Heart.

It is by no means uncommon to hear people complain of pains in the region of the heart, resembling rheumatic or neuralgic affections, and which are too frequently set down by inattentive practitioners as organic diseases.

"Sometimes these pains are confined to this spot, but frequently they extend at the same time, or vicariously, over a greater or less portion of the lungs and stomach. Sometimes they exist simultaneously in the superficial cervical plexus, and extend along the tract of the branches supplied by this to the anterior parts of the thorax; still more frequently, at the very time they are felt most severely in the heart, they shoot with corresponding violence along the nerves of the axillary plexus, and more particularly along the brachial nerve to the elbow, and sometimes as far as the fingers. When this is the case, the affection is confounded with a nervous disease which, during the last twenty years, has been the object of much discussion, and seems to me only a variety of the neuralgia in question. This disease is the

*angina pectoris*, which is very remarkable, and very distressing, when it exists in a high degree, but which is far from possessing the degree of severity attributed to it by many authors."

Laennec first describes what has been called angina pectoris, before he discusses the English pathology of the disease—namely, change of structure in the heart. The following concise description of this dreadful disease is deserving of record.

"The attack commences with a sense of pain, pressure or constriction in the cardiac region or at the end of the sternum. There is at the same time a numbness, occasionally attended with pain in the left arm; rarely in both arms or in one half the body; more rarely still in the right arm only; and sometimes in all the limbs. The painful sensation is particularly felt on the inner side of the arm, as low as the elbow; and sometimes, as already mentioned, it shoots still further down. It is not unusual for the patient to suffer, at the same time, from pains over the fore part of the left chest; and in the female, these sometimes so affect the mamma that the slightest pressure becomes painful. Sometimes, particularly when the paroxysm is severe but short, the patient feels as if the same parts were pierced by iron nails or the claws of an animal. There are also pains in different points of the chest, dyspnœa (in extreme cases suffocative orthopnœa,) violent palpitations, congestion of blood in the head, and sometimes syncope or convulsions. When the attack is over, the patient merely retains a slight feeling of these various symptoms, particularly the numbness of the limbs, the left more especially."

It is well known, that Heberden and Parry attributed this peculiar disease to ossification of the coronary arteries—and this opinion has been embraced by several others. Nothing, however, can be more erroneous than this doctrine. Not one case in ten will be found to present this alteration—and, what is more, the symptoms of angina pectoris are seldom present in those cases where the ossification is found. The general belief in England, Italy, and Germany, is, that the said train of symptoms is dependent on some organic lesion of the heart—that the disease is almost always fatal. Laennec is of a different opinion.

"Angina pectoris, in a slight or middling degree, is extremely common, and exists very frequently in persons who have no organic affection of the heart or large vessels. I have known many individuals who had suffered a few very severe but short attacks of it, and had had no further return of it. I am even of opinion that the prevalent type of disease influences its development, as I have some years met with it frequently, and hardly at all in others. On the other hand, it is certainly true that this affection frequently coincides with organic diseases of the heart; but nothing proves even then that it depends upon such diseases, inasmuch as they are of various kinds, and as the angina exists without any of them. I have examined several subjects who had laboured under this disease, and in whom there coexisted either hypertrophy or dilatation of the heart; and in none of these did I find the coronary arteries ossified. One of these died suddenly during an attack of angina; and such a result need not surprise us, when so severe a nervous affection coexists (as in this case) with extensive hypertrophy. Dr. Desportes, in a dissertation published some years since, has stated opinions very analogous to mine, respecting the nature and seat of this affection: he considers its site to be in the pneumo-gastric nerve. I conceive that the site of the disorder may vary, according to circumstances. For instance, when there exists, at the same time, pain in the heart and lungs, we may presume that the affection is principally seated in the pneumo-gastric; on the other hand, when there is simply a sense of stricture of the heart, without pulmonary pain or much difficulty of breathing, we may consider its site to be in the nervous filaments which the heart receives from the grand sympathetic. Other nerves are also simultaneously affected, either by sympathy or from direct anastomosis; for example, the branches of the brachial plexus, particularly the cubital, are almost always so; the anterior thoracic originating in the superficial cervical plexus, are also frequently affected; and this is also sometimes the case with the branches derived from the lumbar and sacral plexuses, as we find the thigh and leg now and then participating in the pain and numbness. I have even seen the affection confined to the right side of the thorax. In this case the pain and numbness extended to the arm, thigh, and spermatic cord of the same side, and the testicle became swollen during the paroxysms. There was scarcely any pain in the region of the heart; but the attacks were attended by severe palpitation, without any sign of organic lesion of the heart."

The character of the symptoms, M. Laennec thinks, confims this opinion. We know that neuralgiæ of the most unequivocal kind, as sciatica and tic doloureux, give rise to the same variety and species of effects as angina does—namely, acute pain, painful torpor, simple numbness along the tract of nerve, and sometimes spasm of the parts to which the nerves are distributed.

It would be useless to discuss the various opinions on this disease, which have been broached by different writers, since the time of Parry and Fothergill. Dr. Forbes is inclined to agree with Hosack, that the disease " most frequently arises from a plethoric state of the blood-vessels—more especially from a disproportionate accumulation of blood in the heart and large vessels." Dr. F. observes also that, "in persons subject to this complaint, in whom no severe organic disease of the heart existed, he has generally found, by auscultation, that the organ was possessed of thin parietes and feeble powers." It would require a very long life, and a very extensive experience, to speak generally, and with much confidence, on the pathology of angina pectoris. Not more than three or four opportunities have occurred in our own practice, of examining, post mortem, those who have fallen victims to the disease. In only two, was there ossification of the coronary arteries, and, in these, there were other organic lesions. In all the cases, there was a flabby, soft state of the muscular structure of the organ, whether or not accompanied by much fat. But we have seen several cases, unaccompanied by dissection, where there were strong reasons to believe that the disease could not be fairly attributable to ossification of the coronary arteries—and we have found this state of vessels in several subjects, where there was no symptom of angina pectoris before death. The impression on our own minds is, that the nerves of the heart are implicated in the pathology of the disease. The wasting and flabby structure of the organ are, in themselves, rather favourable to this doctrine. We see the muscles of a limb waste and become flaccid, where neuralgia, for example, sciatica, has long obtained. In short, wherever PAIN is a prominent symptom in any complaint, we have a fair right to conclude, that the nervous system of the organ is implicated in the pathology. That the symptoms included under the term angina pectoris may proceed from other causes than affection of the nerves, we will not deny—or at least that various organic derangements may be found after death; but, as the paroxysms come on like those of apoplexy, at various intervals, the organic change necessarily remaining the same, it is reasonable to infer, that the ostensible change of structure detected by the scalpel is rather the predisposing, than the direct occasional cause of the paroxysm. A determination of blood to the head, where there is disease of structure in the brain, will bring on the attack of apoplexy—and so a neuralgia may induce a paroxysm of angina pectoris, where there is already some defective structure in the part. The following therapeutical extract is rather curious.

"The means which I (Laennec) have found most successful in relieving neuralgia of the heart, whether existing in so violent a degree as to be named angina pectoris, or only under the form of slight pains confined to the heart, are those formerly mentioned in the case of neuralgia of the lungs, and especially the magnet. This I use in the following manner: I apply two strongly-magnetized steel plates, of a line in thickness, of an oval shape, and bent so as to fit the part,—one to the left precordial region and the other exactly opposite on the back, in such a manner that the magnetic current shall traverse the affected part. This method is not infallible, any more than others employed in nervous cases; but I must say that it has succeeded better in my hands in the case of angina than any other, as well in relieving the paroxysm, as in keeping it off. Magnetism was, perhaps, too much cried up by some medical men in the last century; but I am of opinion that it is too much neglected at present. That it acts on the animal system, is sufficiently proved by the fact of its giving rise not only to very obvious general effects, but even to local ones. In the case in question, after a certain time it most commonly produces an eruption of small pimples, which are sometimes so painful as to oblige us to interrupt the process for some days. This effect cannot be attributed to the action of the oxidized plates on the skin, as the eruption almost always takes place under the anterior one: and I have observed similar results from plates applied over the abdomen and loins. By means of these plates (applied to the epigastrium and spine) I stopped, at once, a hiccup which had lasted three years. At the end of six months, the patient having one morning neglected to put on the plates, the hiccup returned; but was removed upon their being replaced. In another case in a patient affected with imperfect paraplegia, without any sign of structural lesion of the spine, and for which moxa had been used without success, I inserted, to the depth of half an inch, a needle near the vertebral column and another into the thigh near the external popliteal nerve, and connected these by means of magnetised rods; and at the very instant of contact, there occurred an involuntary dejection, which had never previously happened to the patient. In the angina when the magnet gives but little relief simply, this is sometimes found to be increased on applying a small blister under the anterior plate. During the paroxysm, if the oppression is considerable, we must bleed the patient, if he is at all plethoric. Leeches applied to the epigastrium or cardiac region sometimes give more relief than venesection; but sometimes their application is impracticable from the extreme agitation of the patient. Derivatives are also beneficial, particularly sinapisms to the lower extremities and blisters to the fore part of the chest; as are also antispasmodic medicines, with the infusion of cherry laurel or digitalis, and also the fetid gums. A mild regimen, with the use of the tepid or cold bath, according to the season, are among the best means for preventing a return of the paroxysm."

As to the magnetic treatment, we cannot say any thing from personal observation. The management of the paroxysm must be very different from the treatment during the intervals. Those who have witnessed a severe attack of this terrible disease, can never forget it. The sufferings of the patient must be dreadful. The respiration is sometimes threatened, and the rattling in the throat induces us to draw blood in order to prevent immediate suffocation. In other instances, the breathing appears but little affected, and a cessation of the circulation seems impending, and we are forced to administer cordials. In almost all cases, anodynes and ether, with camphor, are necessary. In the intervals, quietude and temperance, with tranquillity of mind, would be the surest prophylactics—where, alas! are these to be found in this world? Those who have not real woes are tormented with imaginary ones—or, at least, woes of their own creating. Within these very few weeks we witnessed a most distressing case of this disease, which has made a strong impression on our minds, and harrowed up the recollection of several other instances of this deadly malady.

The patient (General B———) was on the borders of 80 years; but remarkably healthy, hale, and vigorous for that advanced age. He was of a very florid complexion, and plethoric constitution—had resided long in a tropical climate—

and was addicted to the pleasures of the table, not amounting perhaps to what might be called intemperance. Up to within a very few weeks of his death (February, 1828) he took active and passive exercise in the open air, and could walk from his residence in Portland-place to the Royal Exchange, as quickly as most men of 50 years of age, though now 80. He had undergone the operation of lithotomy some 20 years ago, under Sir Astley Cooper, and had no return of stone. He never made any complaint of heart affection, and had a strong, equal, and excellent pulse. His only complaint was a sense of occasional fulness about the head, for which he often resorted to cupping. He had also some slight dyspeptic symptoms, in the shape of acidity—depression of spirits—irritability of temper. A very few weeks before his death he complained of shortness of breath, and pain darting from the region of the heart down along the left arm, when ascending the stairs of his own house—and, latterly, when walking in the street, especially if he went against a current of wind. The writer of this article was consulted, and on strict examination, could detect no change of structure or irregularity of action in the heart. He readily recognized, however, that the patient had symptoms of angina pectoris, and stated this to his friends. He advised quietude, temperance, and abstinence from all active exercise. But neither the General nor his friends would be quiet—and therefore they summoned a celebrated surgeon, whose knowledge of *anatomy* must, of course, enable him to detect the most obscure diseases of internal parts. He came—saw disorder of the "digestive organs"—and prescribed blue pill at night, and black draught in the morning. Six days after this treatment had been put in force, the writer was summoned, in the middle of the night, to the patient, who was said to be dying. When he arrived, the General was labouring under one of the most terrific paroxysms of angina pectoris which he ever witnessed. The face was pale, the lips blue, the countenance indicative of unutterable anguish, the pulse scarcely perceptible, the breathing laborious, with what has been not inaptly termed, the "dead rattles" in the throat. The unfortunate sufferer was propped up in bed—tossing from side to side—praying for relief from the horrible pain in the region of the heart and left arm. It was evident that the lungs were gorged with blood, and a lancet was immediately pushed into a vein in the arm. At first a little black blood trickled out—then it came more freely—and, at last, in a stream. When twelve or fifteen ounces of blood were abstracted, the "dead rattles" ceased—the pulse rose—and relief was considerable, though by no means complete. Thirty drops of Battley's liquor opii sedativus, which happened to be in the house, were given—and, in an hour, the patient fell asleep. Next morning, all urgent symptoms were gone. He was ordered to keep his bed for two or three days—and then only sit up in his bed-room. No symptom of angina pectoris returned—and on the sixth or seventh day, the General would no longer submit to restraint. He came down stairs—dined in the parlour on fish—took his first glass of wine in good spirits—and, while drinking the second glass, he died as instantaneously, as if a cannon ball had passed through his chest!

God forbid that we should attribute any part of this tragic finale to the treatment on which the patient was placed for disorder of the "DIGESTIVE ORGANS;" but we can only say that, if General B. died of this said disorder, we are totally ignorant of the nature of the complaint.

We shall take up the subject of other nervous affections of the heart in our next Number.

